# Artificial intelligence computer-aided detection enhances synthesized mammograms: comparison with original digital mammograms alone and in combination with tomosynthesis images in an experimental setting

**DOI:** 10.1007/s12282-022-01396-4

**Published:** 2022-08-24

**Authors:** Takayoshi Uematsu, Kazuaki Nakashima, Taiyo Leopoldo Harada, Hatsuko Nasu, Tatsuya Igarashi

**Affiliations:** 1 Department of Breast Imaging and Breast Intervention Radiology, Shizuoka Cancer Center Hospital, 1007 Shimonagakubo, Nagaizumi, Shizuoka, 411-8777 Japan; 2 Department of Radiology, Hamamatsu University School of Medicine, Shizuoka, Japan; 3 Department of Radiology, Fujieda Municipal General Hospital, Shizuoka, Japan

**Keywords:** Synthesized mammography, Digital breast tomosynthesis, Digital mammography, Artificial intelligence, Breast cancer

## Abstract

**Background:**

It remains unclear whether original full-field digital mammograms (DMs) can be replaced with synthesized mammograms in both screening and diagnostic settings. To compare reader performance of artificial intelligence computer-aided detection synthesized mammograms (AI CAD SMs) with that of DM alone or in combination with digital breast tomosynthesis (DBT) images in an experimental setting.

**Methods:**

We compared the performance of multireader (*n* = 4) and reading multicase (*n* = 388), in 84 cancers, 83 biopsy-proven benign lesions, and 221 normal or benign cases with negative results after 1-year follow-up. Each reading was independently interpreted with four reading modes: DM, AI CAD SM, DM + DBT, and AI CAD SM + DBT. The accuracy of probability of malignancy (POM) and five-category ratings were evaluated using areas under the receiver operating characteristic curve (AUC) in the random-reader analysis.

**Results:**

The mean AUC values based on POM for DM, AI CAD SM, DM + DBT, and AI CAD SM + DBT were 0.871, 0.902, 0.895, and 0.909, respectively. The mean AUC of AI CAD SM was significantly higher (*P* = 0.002) than that of DM. For calcification lesions, the sensitivity of SM and DM did not differ significantly (*P* = 0.204). The mean AUC for AI CAD SM + DBT was higher than that of DM + DBT (*P* = 0.082). ROC curves based on the five-category ratings showed similar proximity of the overall performance levels.

**Conclusions:**

AI CAD SM alone was superior to DM alone. Also, AI CAD SM + DBT was superior to DM + DBT but not statistically significant.

## Introduction

Images for digital breast tomosynthesis (DBT) have already been disseminated for both screening and diagnostic settings. There is evidence of an improved overall performance with digital mammograms (DMs) along with DBT compared to DM alone [1–3]. DM combined with DBT implies double radiation doses, and an additional acquisition time for getting DM. The double radiation dose is not acceptable for screening asymptomatic women, hence, the emergence of synthesized mammograms (SMs) in response to these challenges. SM is generated from DBT data to prevent additional radiation dose from getting two-dimensional DM. Abolishing DM can reduce patients’ compression time, while also reducing their motion, thus ensuring a better wellbeing. Additionally, SM maintains the benefits of DBT by accentuating the data volume on the single two-dimensional SM data. Three systemic reviews show that SM + DBT can replace DM + DBT in screening [1, 4, 5]. Also, several studies have successfully shown that SM alone as non-inferior to DM alone in a diagnostic setting [6–8]. Recently, two studies reported that SM was superior to DM on calcification lesions [9, 10]. However, it remains unclear whether DM can be replaced with SM in both screening and diagnostic settings due to the lower spatial and contrast resolution in the latter, especially for detecting ductal carcinoma in situ (DCIS) with calcification alone [11, 12].

The rate of technological advancement has accelerated exponentially [13]. The rapid rise in technological innovations in medical imaging is capable of solving the problems of SM quality, thereby meeting radiologists’ demands. Previous studies have reported that the use of artificial intelligence computer-aided detection (AI CAD) SM can automatically enhance suspicious findings in the DBT data, thereby improving the quality of SM [14–16]. We hypothesized that AI CAD would improve the quality of images better than that obtained from the DM, with superior performance among the radiologists. This study was undertaken to assess reader performance of AI CAD SM compared with the original full-field DM alone or along with DBT in an experimental setting.

## Materials and methods

### Study design

This retrospective, fully crossed, fully randomized multireader multicase study was approved by the Institutional Review Board of our hospital. All patients were recruited prospectively and gave written informed consent to participate in the study. FUJIFILUM (Tokyo, Japan), which invented AI CAD SM for the reading study, provided the equipment and support for this study. The data and materials submitted for publication were always under the control of the researchers who were not FUJIFILM personnel. According to the joint research and development agreement, 500 DM + DBT examinations with a bilateral two-view (cranio-caudal/mediolateral oblique CC/MLO) image were performed at Shizuoka Cancer Center Hospital between January 2020 and August 2020. All DM + DBT examinations were performed with a narrow-angle DBT system with 15-degree tube motion from 15 projection images (AMULET Innovality; FUJIFILM). DM and DBT were performed during a single breast compression per view. We decided that the comparison of the reader performance was performed at the per-breast level using only mediolateral oblique (MLO) view images taking the reading time and readers’ attention span during the reading study into consideration.

### Case selection

We excluded patients with past surgical history from a case set and developed the case set of 388 breast images from 198 DM/AI CAD SM + DBT examinations in sequence based on the diagnostic work-up patients, which comprises 84 breast cancers, 83 biopsy-proven benign lesions, and 221 normal or benign cases (BI-RADS score of 1 or 2) with negative results after 1-year follow-up. We included dataset with anonymized images and patient’s data with pathology results if an image-guided biopsy and/or surgery was performed. The distribution and mammographic findings were 83 masses (49 malignant and 34 benign), 16 masses with associated calcifications (15 malignant and 1 benign), 5 focal asymmetry densities (2 malignant and 3 benign), 10 architectural distortions (4 malignant and 6 benign), and 53 calcifications (14 malignant and 39 benign). The lesion size of the malignant cases with mass type, ranged from 6 to 42 mm (median 19 mm; mean 20 mm). The verified cancer cases included 71 cases of invasive ductal carcinoma, 8 cases of DCIS, 3 cases of invasive lobular carcinoma, 1 case of tubular carcinoma, and 1 case of invasive micropapillary carcinoma. The breast density distribution ratings of the cases were 206 of 388 (53%) non-dense breasts (including almost entirely fatty or scattered fibroglandular densities) and 182 of 388 (47%) dense breasts (including heterogeneously dense or extremely densities), indicating the tissue density BI-RADS score [17]. The age of the study cohort ranged from 23 to 88 years (median 52 years; mean 54 years).

### Generation of AI CAD SM

We utilized the latest version (under development) of FUJIFILUM image processing system to generate the AI CAD SM used in this study. The AI algorithm uses a fast and efficient convolutional neural network, ESPNet [18]. The development and configuration of the algorithm were completed prior to the study as below. It was trained offline in a data-driven manner by using an expertly annotated tomosynthesis image dataset collected independently, meaning that none of the study cases were used to develop or train the algorithm. The training dataset has 98,098 images belonging to 2548 patients. The data augmentation approach, such as rotation, zoom, and changes in brightness and contrast, was used to enlarge the training dataset. Unlike conventional CAD systems, the AI CAD system acquired knowledge necessary for lesion detection directly from the provided training data and did not rely on explicit encoding or replication of human expert decision processes.

AI CAD SM has improved different finding patterns, such as soft tissue densities, linear structures, and calcifications, detected and extracted from DBT data using AI CAD-like technology. They are new pixel-based synthesis algorithm that merges the selected DBT plane based on AI CAD detections from the DBT data. The lesions detected by AI CAD are not marked or outlined on AI CAD SM.

### Image readers

Four Japanese radiologists who were ranked as having an “A” certification from the Japan Central Organization on Quality Assurance of Breast Cancer Screening (i.e., achieved the scores required for a DM reading instructor in Japan) performed the image evaluation. Readers had between 5 and 30 years of experience in mammography (median, 12 years) and their experience with DBT ranged from 2 to 8 years (median, 4 years). None of the radiologists had prior experience in AI CAD SM reading. Readers did not know the proportion and types of cases (normal, benign, or malignant) or numbers of cases with positive and negative findings.

### Evaluation by the image readers

To acquaint themselves with the workstation and interpretation procedure, all radiologists were trained to review a set of 100 DM + DBT and a set of 100 AI CAD SM + DBT (not included in this study). The radiologists used a reading dual-monitor workstation with an electronic reporting system (Climb-Mammography WS; Climb Medical Systems) and a 5MP DB/DBT-certified diagnostic color display (CCL-S500; JVCKENWOOD) calibrated to the DICOM Grayscale Standard Display Function, for the image interpretation.

The evaluation of the image by the readers was conducted in two stages. Examinations were read twice as follows: (a) DM followed by DM + DBT, and (b) AI CAD SM followed by AI CAD SM + DBT. At least 4-week interval was allowed as a wash-out period. In each mode of reading, radiologists were prompted to rate each breast with the two-dimensional image alone first followed by the DBT rating using the five-point forced BI-RADS (1, 2, 3, 4, or 5) scores. A BI-RADS score of 3 or higher would require the reader to provide the specific type of abnormality and probability of malignancy (POM) rating on a scale of 0–100. On the electronic reporting system, the most suspicious finding was marked and rated.

### Determination of the reference standard

An unblinded review of every case per breast was performed by one radiologist (T.U., a breast imaging radiologist with 30 years of experience in mammogram interpretation and 10 years of experience in tomosynthesis image interpretation), who was not involved in the observer study, to determine the standard pathologic reference and reference mammographic findings and scores. The final morphological feature and Breast Imaging Reporting and Data System (BI-RADS) score [17] were determined by DM. The number of true and false interpretations by the readers was assessed for each image, including the location and radiological characterization of cancers and benign lesions as well as confirmed normal status. Because this study was based on the diagnostic work-up patients, the readers’ final assessments of the images were categorized into two: positive (BI-RADS score of 4–5) and negative (BI-RADS score of 1–3).

### Data analysis

We analyzed the overall accuracy of the breast-based assessment of 388 breasts in 194 patients by four radiologists using two sequential reading modes. Two primary comparisons were performed, namely, (a) DM alone vs. SM alone and (b) DM + DBT vs. AI CAD SM + DBT. The mean area under the receiver operating characteristic (ROC) curve (AUC) was used to assess the accuracy of the reported POM ratings. The reader-averaged AUC was analyzed using the random-reader random-case model to account for the possible correlation in the evaluation of breasts of the same patients. We also analyzed the correlation between repeated assessments of the same cases by different radiologists using different modalities, and between-reader variability. Bootstrap percentile confidence intervals and the corresponding estimated *P* values were computed based on 100,000 bootstrap samples (1000 resamples of cases by 100 resamples of readers). In the secondary analysis, we analyzed ROC curves obtained from the forced BI-RADS ratings. Statistical inferences for all analyses were performed at the significance level of 0.05. The statistical analyses were performed using the R package for Windows (version 4.0.2, R Foundation for Statistical Computing, Vienna, Austria.).

## Results

### General findings

The mean (± standard deviation) compressed breast thickness during DM + DBT was 38.8 mm ± 14.5. The mean glandular dose for a single mammographic view was 1.12 mGy ± 0.32 (standard deviation) for DM and 1.62 mGy ± 0.49 for DBT. These dose levels constitute an average dose reduction of 41% (1.12 mGy/2.74 mGy) for AI CAD SM + DBT as compared with DM + DBT.

All readers completed the reading sessions as planned; 3104 case reports were received (388 × 4 × 2), and there were no missing data.

### The mean AUCs for the reading modes

**Fig. 1:**
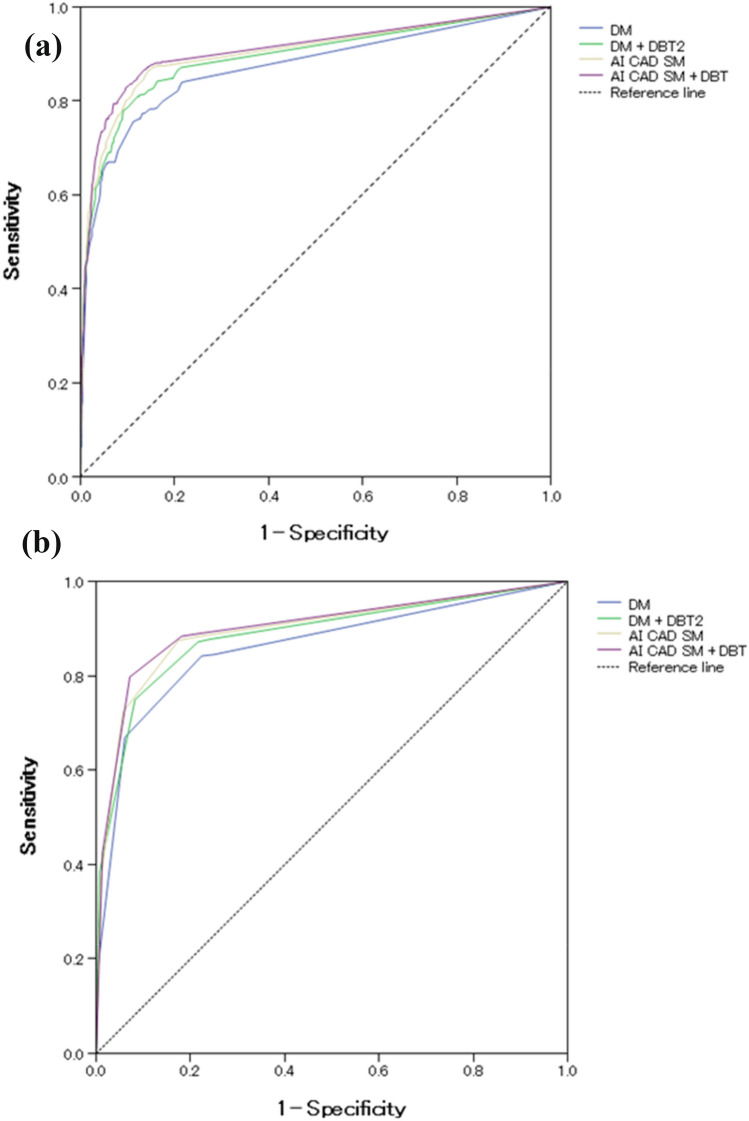
**a** Overall ROCs curve based on probability of malignancy ratings for individual breasts. **b** Overall ROCs curve based on five-point forced BI-RADS scores ratings for individual breasts

The mean AUC values based on POM for DM and AI CAD SM alone were significantly different with 0.871 and 0.902, respectively (difference 0.03; 95% confidence interval [CI] 0.012 and 0.051; *P* = 0.002, Fig. 1a). The AUC of AI CAD SM alone was significantly higher than that of DM alone. For DM + DBT and AI CAD SM + DBT, AUCs were 0.895 and 0.909, respectively (difference 0.016; 95% CI − 0.001 and 0.033; *P* = 0.082). The AUC of AI CAD SM + DBT was higher that of DM + DBT, but the difference was not statistically significant. All four readers performed somewhat better with AI CAD SM than with DM (Table 1), and three readers performed somewhat better with AI CAD SM + DBT than with DM + DBT (Table 1). Additionally, the mean AUC of AI CAD SM alone and DM + DBT did not differ significantly (*P* = 0.356), and three readers performed somewhat better with AI CAD SM alone than with DM + DBT (Table 1). Except for reader 4, who performed slightly worse (AUC difference 0.004) with DM + DBT compared to DM alone, all other readers improved in their performance from the corresponding sequential reading modes when DBT was made available to them after a review of either the DM or AI CAD SM alone.

**Table 1:** AUC based on POM ratings and forced BI-RADS ratings for each reader for breast-level analysis

Reader	AUC based on POM ratings				AUC-based forced BI-RADS ratings			
	DM	AI CAD SM	DM + DBT	AI CAD SM + DBT	DM	AI CAD SM	DM + DBT	AI CAD SM + DBT
1	0.863	0.912	0.892	0.917	0.857	0.908	0.892	0.916
2	0.899	0.902	0.922	0.908	0.882	0.890	0.913	0.892
3	0.843	0.913	0.890	0.925	0.834	0.910	0.884	0.922
4	0.878	0.881	0.874	0.886	0.870	0.877	0.864	0.883
Mean	0.871	0.902	0.895	0.909	0.861	0.896	0.888	0.903

*DM* digital mammogram, *AI CAD SM* artificial intelligence computer-aided detection enhanced synthesized mammogram, *DBT* digital breast tomosynthesis images

ROC curves based on the forced BI-RADS ratings (Fig. 1b) showed similar proximity of the overall performance levels (Table 1).

### Cancer diagnostic sensitivities with reading modes

Cancer diagnostic sensitivities for DM, AI CAD SM, DM + DBT, and AI CAD SM + DBT were 67.0%, 72.3%, 75.0%, and 79.8%, respectively (Table 2). The radiologists significantly improved their cancer diagnostic sensitivity when reading AI CAD SM compared to DM either alone or in combination with DBT (difference 0.054; 95% CI 0.015 and 0.094; *P* = 0.008, for alone; difference 0.048; 95% CI 0.012 and 0.083; *P* = 0.006, for in combination with DBT). The specificities for DM, AI CAD SM, DM + DBT, and AI CAD SM + DBT were 94.0%, 94.4%, 91.8%, and 92.8%, respectively. The specificity was maintained regardless of AI CAD SM or DM.

**Table 2:** Sensitivity and specificity of each reading mode for overall and each finding

	DM (%)	AI CAD SM (%)	DM + DBT (%)	AI CAD SM + DBT (%)
All (*n* = 388)				
Sensitivity	67.0	72.3	75.0	79.8
Specificity	94.0	94.4	91.8	92.8
Non-dense breast (*n* = 206)				
Sensitivity	72.1	77.4	81.7	84.6
Specificity	93.8	94.0	92.8	93.2
Dense breast (*n* = 182)				
Sensitivity	58.6	64.1	64.1	71.9
Specificity	94.0	94.7	90.8	92.5
Soft tissue density lesion (*n* = 104)				
Sensitivity	66.7	74.2	75.0	82.2
Specificity	80.3	84.2	77.6	82.2
Calcification lesion (*N* = 53)				
Sensitivity	80.4	73.2	85.7	76.8
Specificity	85.3	85.9	83.3	85.3

*DM* digital mammogram, *AI CAD SM* artificial intelligence computer-aided detection enhanced synthesized mammogram, *DBT* digital breast tomosynthesis images

### Non-dense breast cases

For non-dense breast cases (*n* = 206), sensitivities for DM, AI CAD SM, DM + DBT, and AI CAD SM + DBT were 72.1%, 77.4%, 81.7%, and 84.6%, respectively (Table 2). The sensitivity of AI CAD SM alone was significantly higher than that of DM alone (difference 0.053; 95% CI 0.000 and 0.106; *P* = 0.038). The sensitivity of AI CAD SM + DBT was higher than that of DM + DBT but not statistically significant (difference 0.029; 95% CI − 0.010 and 0.067; *P* = 0.114).

### Dense breast cases

For dense breast cases (*n* = 182), sensitivities for DM, AI CAD SM, DM + DBT, and AI CAD SM + DBT were 58.6%, 64.1%, 64.1%, and 71.9%, respectively. The sensitivity of AI CAD SM alone was higher than that of DM alone but not statistically significant (difference 0.055; 95% CI − 0.016 and 0.125; *P* = 0.082). The sensitivity of AI CAD SM + DBT was significantly higher than that of DM + DBT (difference 0.078; 95% CI 0.008 and 0.148; *P* = 0.018).

### Soft tissue density and distortion lesions

**Fig. 2:**
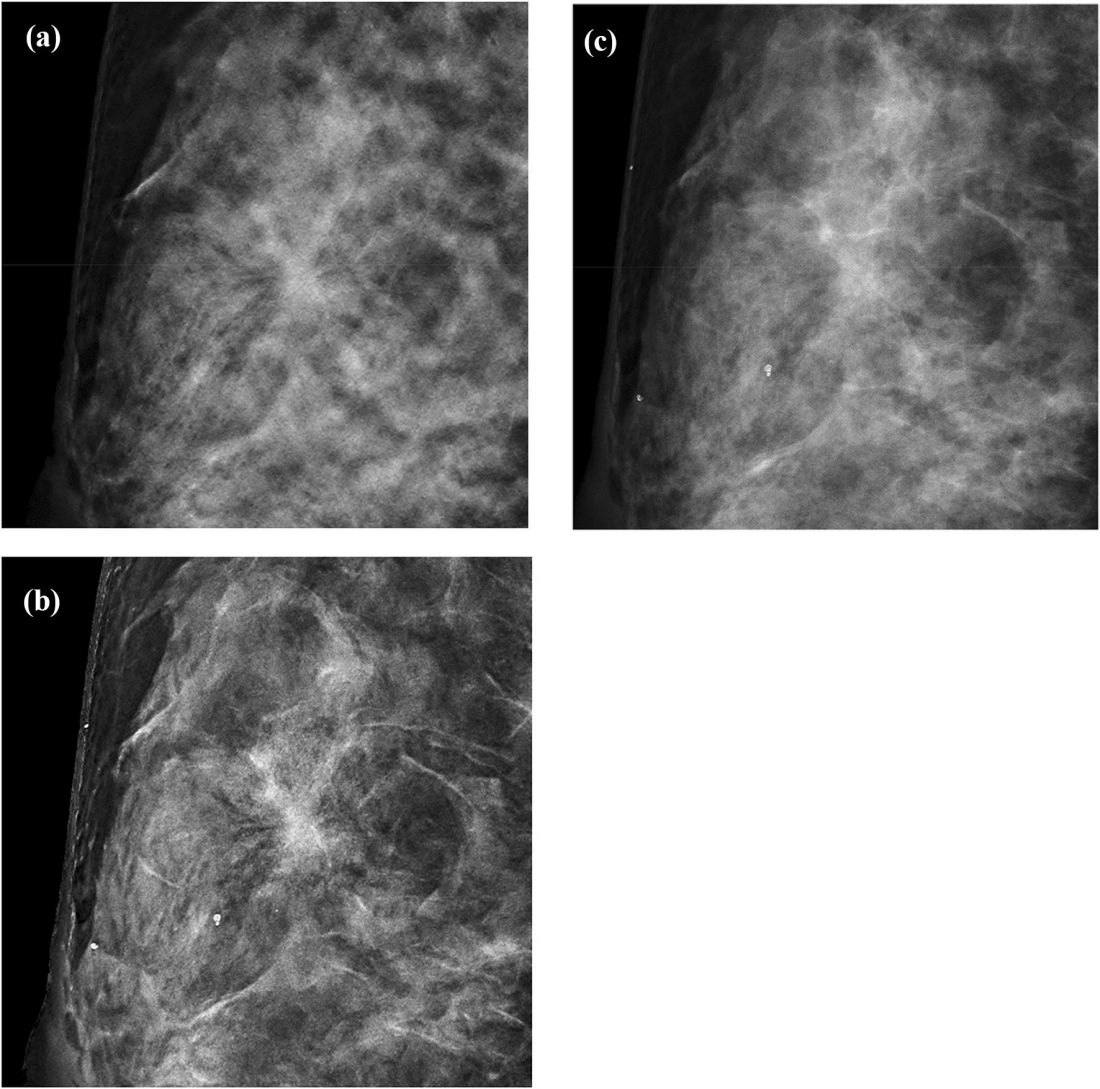
Image in a 53-year-old woman with extremely dense breasts proved to be invasive lobular carcinoma at surgery. **a** DBT shows a 10 mm architectural distortion. **b** AI CAD SM also demonstrates the architectural distortion, but **c** DM fails to depict it

For soft tissue density lesions including mass, focal asymmetry density, and mass + calcification (*n* = 104), the sensitivities for DM, AI CAD SM, DM + DBT, and AI CAD SM + DBT were 66.7%, 74.2%, 75.0%, and 82.2%, respectively (Table 2). The radiologists significantly improved their cancer diagnostic sensitivity when reading the AI CAD SM compared to DM either alone or in combination with DBT (difference 0.076; 95% CI 0.030 and 0.125; *P* < 0.001, for alone; difference 0.072; 95% CI 0.034 and 0.117; *P* < 0.001, for in combination with DBT). For distortion lesions (*n* = 10), malignant histologic findings in these four lesions were DCIS in one, invasive ductal carcinoma in two, and invasive lobular carcinoma in one. The 10 mm invasive lobular carcinoma was detected by three readers with AI CAD SM but not detected by all four readers with DM (Fig. 2).

### Calcification lesions

**Fig. 3:**
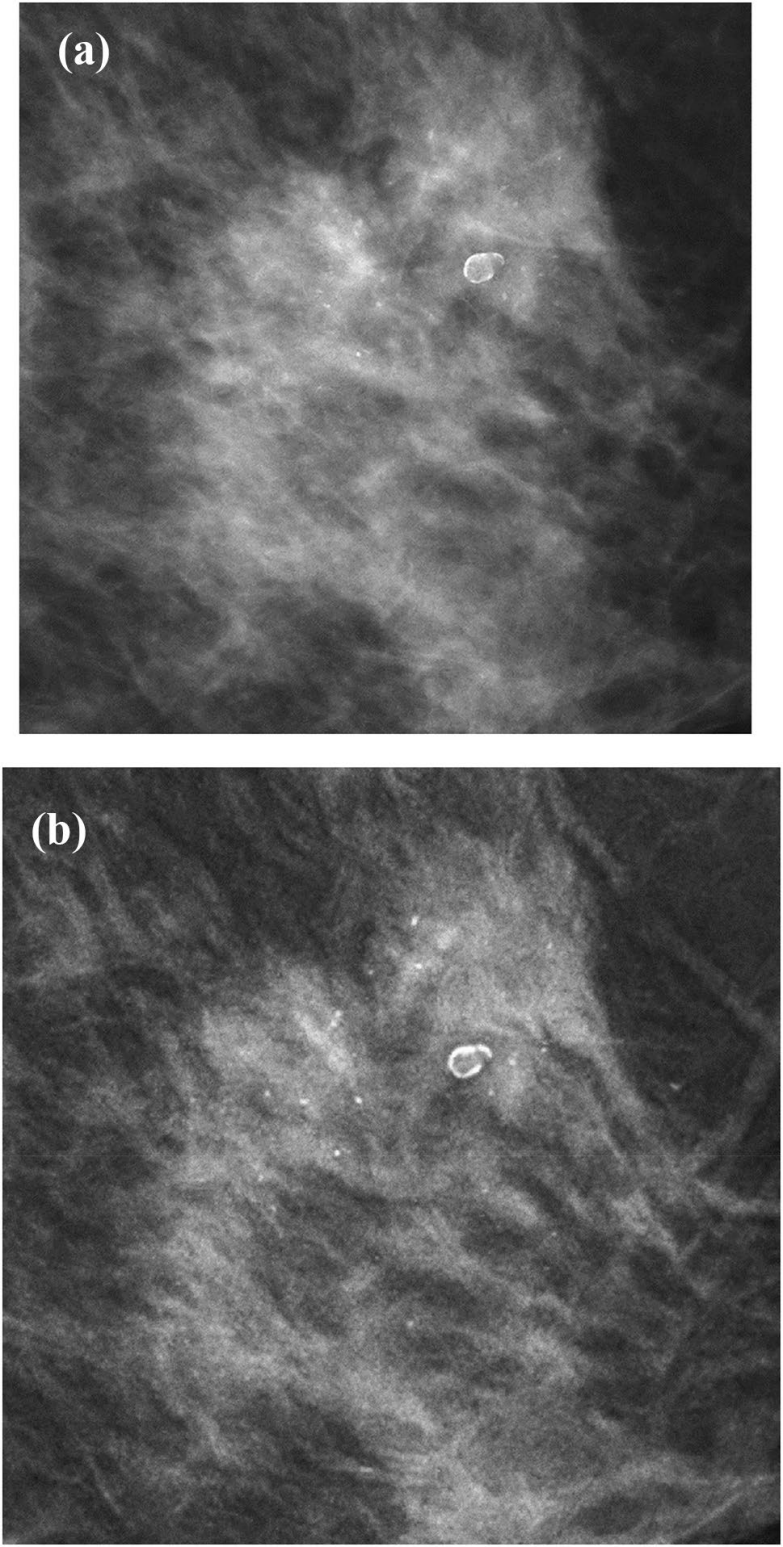
Image in a 52-year-old woman with scattered fibroglandular breasts proved to be DCIS at surgery. **a** DM shows 31 mm segmental amorphous calcifications. The mean BI-RADS score was 3.75. **b** AI CAD SM demonstrates it as grouped round calcifications if anything. However, the visualization is better than that of DM. The mean BI-RADS score was also 3.75

For calcification lesions (*n* = 53), sensitivities for DM, AI CAD SM, DM + DBT, and AI CAD SM + DBT were 80.4%, 73.2%, 85.7%, and 76.8%, respectively (Table 2). The sensitivity of AI CAD SM alone was lower than that of DM alone but not statistically different (difference − 0.071; 95% CI − 0.161 and − 0.018; *P* = 0.204) (Fig. 3). The sensitivity of AI CAD SM + DBT was significantly lower than that of DM + DBT (difference − 0.089; 95% CI − 0.161 and − 0.018; *P* = 0.004). In calcification morphology, the mean BI-RADS scores of AI CAD SM tend to be lower than those of DM, especially for amorphous calcifications (Table 3).

**Table 3:** Characteristics of malignant calcification lesions and mean BI-RADS scores of four readers comparing DM and AI CAD SM

Calcification morphology	Calcification distribution	Size (mm)	Mean BI-RADS score	
			DM	AI CAD SM
Amorphous	Grouped	14	2.5	1.5
Amorphous	Grouped	31	3.75	3.75
Amorphous	Grouped	10	3.75	3.5
Amorphous	Grouped	14	4.25	4
Coarse heterogeneous	Grouped	13	3.5	3.25
Coarse heterogeneous	Grouped	16	4	3.75
Coarse heterogeneous	Grouped	19	3.75	4
Coarse heterogeneous	Grouped	18	4	4
Coarse heterogeneous	Segmental	60	4.5	4.5
Coarse heterogeneous	Segmental	30	5	4.5
Coarse heterogeneous	Segmental	33	4.5	4.25
Fine pleomorphic	Segmental	20	4.75	5
Fine pleomorphic	Segmental	24	3.5	4.25
Fine pleomorphic	Segmental	30	4.75	4.5

*DM* digital mammogram, *AI CAD SM* artificial intelligence computer-aided detection enhanced synthesized mammogram, *BI-RADS* breast imaging reporting and data system

## Discussion

In this study, we evaluated diagnostic performance of AI CAD SM vs. original DM either alone or in combination with DBT and compared the performance of multireader reading multicase for breast density, soft tissue density lesions, and calcification lesions. The mean AUC values based on POM for DM, AI CAD SM, DM + DBT, and AI CAD SM + DBT were 0.871, 0.902, 0.895, and 0.909, respectively. The improving diagnostic performance of AI CAD SM compared to DM led to an improved sensitivity, especially for non-calcification lesions. Our results showed that when used alone, AI CAD SM could yield better diagnostic performance than to that of DM, although previous studies reported SM to be non-inferior to DM [6–8]. These results agreed with two studies [9, 10], even though this study was conducted using a large-scale study design. This study also showed that the latest version of the image processing algorithm used to generate the AI CAD SM might attain the level required by radiologists. Additionally, our finding is unique to show that the diagnostic performance of AI CAD SM was not inferior to that of DM + DBT. There was no statistically significant difference although the combined AI CAD SM and DBT had better diagnostic performance than that of AI CAD SM used alone. These results imply that AI CAD SM maintains the performance benefits of DBT examinations, and AI CAD SM can be used as standalone 2D mammograms in a diagnostic setting. This can become useful in clinical practice to address the issues regarding reduced reading time and radiation dose. This study also showed that AI CAD SM + DBT were superior to DM + DBT even though it was not significant. Therefore, AI CAD SM + DBT can be considered acceptable and adequate for routine clinical practice.

Evaluation of the performance of the readers on breast density is also an important research point. This study showed the sensitivity of AI CAD SM was higher than that of DM in dense breast cases. Comparable results were obtained in several other studies conducted on SM [7, 8, 19–22]. SM, including AI CAD SM, generated from DBT data has power of eliminating the overlapping breast tissue resistance from dense breasts. Thus, we may proactively use AI CAD SM for women with dense breasts.

Regarding soft tissue density lesions including mass, focal asymmetry density, and mass + calcification, the radiologists significantly improved their cancer diagnostic sensitivity when reading the AI CAD SM compared to DM either alone or in combination with DBT. This is because AI CAD SM maintains the performance benefits of DBT examinations. Subtle spiculations that is obscured by glandular tissue on a 2D mammogram is better seen on the AI CAD SM (Fig. 2). AI CAD SM is good at detecting subtle spiculations and distortions.

This study also showed that AI CAD SM was not significantly different from DM for diagnostic sensitivity of calcification lesions. In the case of malignant calcification, the mean BI-RADS scores of AI CAD SM tend to be lower than those of DM. However, the calcification conspicuity of AI CAD SM is better than that of DM (Fig. 3). All the malignant calcification lesions, excluding one amorphous calcification, had a mean BI-RADS score 3 or above. If it were in a screening setting, they would be detected and recalled because of score 3 or above as positive.

Our results might imply that AI CAD SM is able to be used as a guide during the interpretation of the DBT. However, it is beyond the scope of this study. There are no data available for the effect of AI CAD SM on clinical performance and patient outcome. Further studies are needed.

The DM + DBT requires a radiation dose that is approximately double that for DM alone. However, the radiation dose level for the combined examination was set to be below limits approved by the U.S. Food and Drug Administration, which constitutes an acceptable risk. In this study, with a mean breast thickness of 38.8 mm, the radiation dose levels for DM + DBT were 2.4 times those for DM alone (2.74 mGy and 1.12 mGy, respectively). This indicated a substantially lower mean glandular dose for AI CAD SM + DBT at 41% of the mean glandular dose for DM + DBT. Comparable results were obtained in several other studies conducted on SM [23]. We believe that the current lower-dose approach of AI CAD SM + DBT is more appropriate for screening purposes.

Our study had several limitations. First, only one experienced radiologist established the reference findings of the study cohort. Consequently, there was a substantial risk of inter-and intra-reader variability in the analysis of the mammographic data, especially for calcification descriptors [24]. Therefore, the reference findings of a single reviewer could have influenced our findings. Second, this study only used the MLO view, which implies that limited access to the CC view could marginally increase the sensitivity and specificity of the reading mode [25]. Therefore, further validation with two views is necessary. Third, this study used a prototype system with a new algorism of AI CAD-like technology. Although some current SMs generated from DBT have been already commonly enhanced with suspected lesions detected by AI CAD-like technology [26], additional validation of the algorism is highly necessary. Finally, this study was conducted using cases and radiologists from a single country and based on a retrospective design at a single institution with a single vendor. Thus, these study characteristics may restrict the generalizability of our findings, necessitating a prospective valuations design with a range of radiologists, patients, and institutions. All identified limitations might have led to some unintentional bias. Therefore, the results should be cautiously considered as preliminary results. Nonetheless, to the best of our knowledge, our study is the first to fully assess readers’ performance when AI CAD SM vs. original full-field DM is either used alone or in combination with DBT in an experimental setting.

In conclusion, AI CAD SM can be selected exclusively over DM due to its superiority to DM alone. Similarly, AI CAD SM + DBT was also superior to DM + DBT although not statistically significant. Moreover, the diagnostic performance of AI CAD SM alone was non-inferior to that of DM + DBT. Thus, it might imply that the new SM should be clinically prioritized over DM.

## References

[1] Alabousi M, Wadera A, Kashif Al-Ghita M, Kashef Al-Ghetaa R, Salameh JP, Pozdnyakov A (2021). Performance of digital breast tomosynthesis, synthetic mammography, and digital mammography in breast cancer screening: a systematic review and meta-analysis. J Natl Cancer Inst.

[2] Alabousi M, Zha N, Salameh JP, Samoilov L, Sharifabadi AD, Pozdnyakov A (2020). Digital breast tomosynthesis for breast cancer detection: a diagnostic test accuracy systematic review and meta-analysis. Eur Radiol.

[3] Ko MJ, Park DA, Kim SH, Ko ES, Shin KH, Lim W (2021). Accuracy of digital breast tomosynthesis for detecting breast cancer in the diagnostic setting: a systematic review and meta-analysis. Korean J Radiol.

[4] Heywang-Köbrunner SH, Jänsch A, Hacker A, Weinand S, Vogelmann T (2021). Digital breast tomosynthesis (DBT) plus synthesised two-dimensional mammography (s2D) in breast cancer screening is associated with higher cancer detection and lower recalls compared to digital mammography (DM) alone: results of a systematic review and meta-analysis. Eur Radiol.

[5] Zeng B, Yu K, Gao L, Zeng X, Zhou Q (2021). Breast cancer screening using synthesized two-dimensional mammography: a systematic review and meta-analysis. Breast.

[6] Zuley ML, Guo B, Catullo VJ, Chough DM, Kelly AE, Lu AH (2014). Comparison of two-dimensional synthesized mammograms versus original digital mammograms alone and in combination with tomosynthesis images. Radiology.

[7] Choi JS, Han BK, Ko EY, Ko ES, Shin JH, Kim GR (2016). Comparison between two-dimensional synthetic mammography reconstructed from digital breast tomosynthesis and full-field digital mammography for the detection of T1 breast cancer. Eur Radiol.

[8] Mariscotti G, Durando M, Houssami N, Fasciano M, Tagliafico A, Bosco D (2017). Comparison of synthetic mammography, reconstructed from digital breast tomosynthesis, and digital mammography: evaluation of lesion conspicuity and BI-RADS assessment categories. Breast Cancer Res Treat.

[9] Murakami R, Uchiyama N, Tani H, Yoshida T, Kumita S (2020). Comparative analysis between synthetic mammography reconstructed from digital breast tomosynthesis and full-field digital mammography for breast cancer detection and visibility. Eur J Radiol Open.

[10] Baldelli P, Cardarelli P, Flanagan F, Maguire S, Phelan N, Tomasi S (2021). Evaluation of microcalcification contrast in clinical images for digital mammography and synthetic mammography. Eur J Radiol.

[11] Durand MA (2018). Synthesized mammography: clinical evidence, appearance, and implementation. Diagnostics (Basel).

[12] Zuckerman SP, Sprague BL, Weaver DL, Herschorn SD, Conant EF (2020). Survey results regarding uptake and impact of synthetic digital mammography with tomosynthesis in the screening setting. J Am Coll Radiol.

[13] Kobayashi Y, Ishibashi M, Kobayashi H (2019). How will "democratization of artificial intelligence" change the future of radiologists?. Jpn J Radiol.

[14] Benedikt RA, Boatsman JE, Swann CA, Kirkpatrick AD, Toledano AY (2018). Concurrent computer-aided detection improves reading time of digital breast tomosynthesis and maintains interpretation performance in a multireader multicase study. AJR Am J Roentgenol.

[15] Balleyguier C, Arfi-Rouche J, Levy L, Toubiana PR, Cohen-Scali F, Toledano AY (2017). Improving digital breast tomosynthesis reading time: a pilot multi-reader, multi-case study using concurrent computer-aided detection (CAD). Eur J Radiol.

[16] James JJ, Giannotti E, Chen Y (2018). Evaluation of a computer-aided detection (CAD)-enhanced 2D synthetic mammogram: comparison with standard synthetic 2D mammograms and conventional 2D digital mammography. Clin Radiol.

[17] American College of Radiology (2013) Breast imaging reporting and data system (BI-RADS), 5th edn. ACR, Reston

[18] Mehta S, Rastegari M, Caspi A, Shapiro L, Hajishirzi H (2018) ESPNet: efficient spatial pyramid of dilated convolutions for semantic segmentation. Comput Sci. https://arxiv.org/abs/1803.06815v3

[19] Mesurolle B, El Khoury M, Travade A, Bagard C, Pétrou A, Monghal C (2021). Is there any added value to substitute the 2D digital MLO projection for a MLO tomosynthesis projection and its synthetic view when a 2D standard digital mammography is used in a one-stop-shop immediate reading mammography screening?. Eur Radiol.

[20] Østerås BH, Martinsen ACT, Gullien R, Skaane P (2019). Digital mammography versus breast tomosynthesis: impact of breast density on diagnostic performance in population-based screening. Radiology.

[21] Lowry KP, Coley RY, Miglioretti DL, Kerlikowske K, Henderson LM, Onega T (2020). Screening performance of digital breast tomosynthesis vs digital mammography in community practice by patient age, screening round, and breast density. JAMA Netw Open.

[22] Rafferty EA, Park JM, Philpotts LE, Poplack SP, Sumkin JH, Halpern EF (2014). Diagnostic accuracy and recall rates for digital mammography and digital mammography combined with one-view and two-view tomosynthesis: results of an enriched reader study. AJR Am J Roentgenol.

[23] Zuckerman SP, Maidment ADA, Weinstein SP, McDonald ES, Conant EF (2017). Imaging with synthesized 2D mammography: differences, advantages, and pitfalls compared with digital mammography. AJR Am J Roentgenol.

[24] Berg WA, Arnoldus CL, Teferra E, Bhargavan M (2001). Biopsy of amorphous breast calcifications: pathologic outcome and yield at stereotactic biopsy. Radiology.

[25] Zackrisson S, Lång K, Rosso A, Johnson K, Dustler M, Förnvik D (2018). One-view breast tomosynthesis versus two-view mammography in the Malmö Breast Tomosynthesis Screening Trial (MBTST): a prospective, population-based, diagnostic accuracy study. Lancet Oncol.

[26] Chan HP, Helvie MA (2021). Using single-view wide-angle DBT with AI for breast cancer screening. Radiology.

